# Neologisms are epidemic: Modeling the life cycle of neologisms in China 2008-2016

**DOI:** 10.1371/journal.pone.0245984

**Published:** 2021-02-03

**Authors:** Menghan Jiang, Xiang Ying Shen, Kathleen Ahrens, Chu-Ren Huang

**Affiliations:** 1 Department of Chinese Language and Literature, Peking University, Beijing, China; 2 Department of Chinese and Bilingual Studies, The Hong Kong Polytechnic University, Hong Kong, China; 3 Department of Physics, Chinese University of Hong Kong, Hong Kong, China; 4 Department of English, The Hong Kong Polytechnic University, Hong Kong, China; 5 HK PolyU-PKU Research Centre on Chinese Linguistics, Hong Kong, China; 6 Research Centre for Professional Communication in English, The Hong Kong Polytechnic University, Hong Kong, China; 7 Shenzhen JL Computational Science And Applied Research Institute, Shenzhen, China; Aix-Marseille Université, FRANCE

## Abstract

This paper adopts models from epidemiology to account for the development and decline of neologisms based on internet usage. The research design focuses on the issue of whether a host-driven epidemic model is well-suited to explain human behavior regarding neologisms. We extracted the search frequency data from Google Trends that covers the ninety most influential Chinese neologisms from 2008-2016 and found that the majority of them possess a similar rapidly rising-decaying pattern. The epidemic model is utilized to fit the evolution of these internet-based neologisms. The epidemic model not only has good fitting performance to model the pattern of rapid growth, but also is able to predict the peak point in the neologism’s life cycle. This result underlines the role of human agents in the life cycle of neologisms and supports the macro-theory that the evolution of human languages mirrors the biological evolution of human beings.

## Introduction

Awareness of the relevance of neologisms to the discovery of new knowledge can be traced back to at least 1871, with a series of letters to *Nature* initiated by Ingleby [1]. More recently, neologisms have become part of our daily life as people now communicate with others, seek information, and provide opinions online. Ubiquitous and immediate access to information with the potential for real-time responses has provided a strong impetus for the creation of ideas and has allowed such ideas to be spread more broadly and more quickly than ever before. Neologisms, or newly coined words, are found in all formats, including both traditional and new media. In fact, research has shown that neologisms nowadays are generated primarily on the internet [2, 3]. Since these neologisms appear online and are immediately archived with a time-stamp, these first uses can be easily identified as milestones of language change and variation [4], unlike the first instance of neologisms in traditional media. However, linguistic research on these “internet neologisms” has not been extensively conducted and a comprehensive theoretical model that can clearly describe a neologism’s life cycle has yet to be proposed, as recent studies tracking language usage have only focused on *post hoc* analyses of behavioral changes over shorter [5] or longer [6] periods of time. Hence, a robust, *a priori* model of the life cycle of neologisms is needed. Such a model will have implications as to how languages change and evolve, as well as how new ideas propagate and influence collective human behavior.

Modeling language evolution is a challenging issue [7–9], and modeling neologisms is no exception [10]. Previous empirical studies on neologisms were driven by lexicographic concerns and focused on research issues such as the life cycle of neologisms or the prediction of which new words would be successfully incorporated into the lexicon of a language [11–14]. A variety of empirical studies also focused on the conditions or factors that could maximize the chances of survival of a new word, i.e., to determine whether a neologism would disappear or become a regular lexical entry [15, 16]. For instance, word frequency and the niche that a new word fills were often considered to be important factors for the survival rate of a new word [17, 18].

Internet neologisms evolve much faster and have the potential to spread more widely than ever before, but at the same time may fade and disappear quickly. Traditional linguistic approaches to date have lacked a way to deal with either the scale or the speed of the life cycles of neologisms. In this article, we propose to leverage the aggregated data from Google Trends, which provides the popularity of a selected word within a flexible range of time intervals. The granularity of the reported data is accurate to each day and the reported popularity is normalized on a scale of zero to one hundred. In this current study, we focus on the neologisms found on the internet in Chinese and selected yearly by experts as strong candidates for lexicalization.

Given the close link between the explosion of neologisms and new media and the view that language is a “culturally transmitted replicator” [19], an obvious model for the life cycle is the memetic model. This approach models the growth and spread of new words, which are sometimes called lexical memes, similar to cultural memes. The metaphor of genetic evolution is also relevant as dominant memetic models are genetic replications (and vice versa [20–25]). That is, these models predict the development of memes in terms of their ability to propagate just like the ability of genes to self-replicate. Such models are often constrained in terms of peak time or other resource variables.

Although most recent studies on neologisms focused on this memetic hypothesis, we found that the memetic model did not provide a good description of the fast rising and decaying pattern of internet neologisms. The memetic model assumes that the evolution of a word lasts a long period, and that the overall survival rate of a meme can be expressed in terms of the meme’s fitness *F*(*m*), which characterizes the ratio between the expected number of memes at the next time step or generation and the average number of memes at the present time [26]. This type of model involves a conventional pattern of exponential growth if *F*(*m*) > 1 (i.e. for a meme to spread), exponential decay if *F*(*m*) < 1, and stability if *F*(*m*) = 1.

Mathematically, the memetic model varies according to the parameter of fitness, which indicates the self-replicating ability of the meme/word. If fitness is larger than one, the word will be judged as a survivor who can continuously replicate itself. If fitness fails to reach this critical value, replication is not sustainable, and the word will fade away. Applying the memetic model to neologisms assumes that the survival of a neologism depends mostly on its ability to continue to replicate. This model further assumes that fitness should be independent of time or remain constant for a long enough time. Hence, the model predicts a monotonic rise or decay of the popularity of the neologism. However, we know as a fact that the fast evolution of internet neologisms does not involve a monotonic growth curve. For a neologism, just like any other word, there must be an inflection point in its life cycle where the growth of the word plateaus or turns downward. Hence, the full life cycle of internet neologisms clearly cannot be expressed by a monotonic function. In order to avoid the monotonic prediction that is obviously incorrect, one has to turn the monotonic memetic model into a piecewise function consisting of several domains with different fitness parameters. However, this step turns the memetic model into *a posteriori* model, as the piecewise fitness parameters are assigned to retrofit known data, which indicates that the model cannot predict the location of the inflection points. Therefore, we argue that a simple memetic model is not suitable for modeling neologisms.

One critical difference between genes and words is that genes are part of a living organism and the sentience of the organisms has no direct bearing on the replication of the genes. Words, on the other hand, exist independently of the speakers as sentient agents, but can only exist through the action of these agents. As the life cycle of neologisms involves both the new words and the speakers who use them, it is also possible to model the life cycle of neologisms in terms of the speaking population. In this respect, this life cycle spread could also be viewed as an epidemic, such as the metaphor of contagion used in [27]. Thus, we propose that the life cycle of neologisms should be better predicted by a host-driven epidemic model, as this type of model is best known for modeling the spreading and growth of a disease over a large population.

Most epidemic models are constructed to account for the competition between the transmissibility of the virus and the infected populations’ ability to recover. As such, an epidemic model intrinsically implies the existence of an inflection point where the number of recovered people exceeds the number of infected ones, or vice versa. Hence, the epidemic model is an *a priori* model that can provide a good overall description of the neologisms’ evolution patterns observed from the data, with a powerful predictive power to locate the inflection point. Therefore, we suggest that it is more suitable for modeling the life cycle of internet neologisms.

The words selected are from the journal 咬文嚼字*Yao3Wen1Jiao2Zi4*, an authoritative journal on lexicology and philology in China. For each year since 2009, 咬文嚼字 *Yao3Wen1Jiao2Zi4* has published a list of the most successful neologisms from the preceding year. The status of these words is similar to Words of the Year selected by the American Dialect Society (https://www.americandialect.org/woty) for being notable, prominent, and characteristic of the discourse in the preceding year [15]. In fact, the selection criteria adopted by 咬文嚼字 *Yao3Wen1Jiao2Zi4* [28] can be shown to closely mirror those of the American Dialect Society: 1) Fashionable: The words are able to reflect the characteristics of the language of the media and social communication in each year (corresponding to the “characteristic of the discourse of the year just past”); 2) Popular: The words are familiar to the people who read the press regularly (corresponding to “Notable”); and 3) Expressive: E.g., being creative, humorous, ironic, vivid, concise, or euphemistic (corresponding to “Prominent”).

Based on the selection by 咬文嚼字 *Yao3Wen1Jiao2Zi4*, we have taken 10 new words of the year from each year between 2008-2016, for a total of 90 new words. We have not taken the more recent neologisms from the past three years to ensure that there is enough longitudinal data for the life cycle of each new word. By modeling the life cycle of these neologisms with the epidemic model, we aim to resolve the following research questions:

1. What is the typical life cycle and what are the most important features of a successful neologism?
2. Does the epidemic model clearly describe and predict the development of neologisms?

At first glance, the majority of successful internet neologisms have a similar development, according to their popularity over time. The life cycle tends to begin with a relatively quick ascent in terms of popularity and then slowly descends after the peak. This general pattern is compatible with a host-driven epidemic model; but it is still necessary to empirically verify whether the epidemic model can satisfactorily describe and predict the life cycle of an internet neologism. In what follows, we describe our studies of modeling the extracted data using a host-driven epidemic model.

This article aims to provide a new method to obtain statistical data of neologisms from internet search engines and utilizes a classical mathematical model to explain variations in a neologism’s popularity.

## Data and methodology

### Data source

This study covers 90 neologisms announced by the journal 咬文嚼字 *Yao3Wen1Jiao2Zi4*, spanning the nine year period from 2008 to 2016 [29–37]. To model the rapidly rising and decay-ing internet neologisms, we measure the prevalence of a word by the number of times it has been searched for on Google. This data sampling choice is based on two important observa-tions: First, a neologism only comes into existence at its coinage and has not been attested on the internet before. Thus, it is reasonable to treat its initial search term uses as evidence for the emergence of the new word.

Second, although it is not possible to rule out dictionary searches among all browser usages, it is reasonable to assume that early dictionary searches of a neologism are prompted by encountering that word in actual usage, such as while browsing for new information or to express a new concept. Hence, it is a *bona fide* use of the word in real context. In other words, we consider that web-searches of a neologism early in its life cycle are predominantly usage driven. The probability of a content or linguistic information search of such a word is negligible as such searches require that the user knows, or knew of this word already, and has some linguistic knowledge about it.

Relevant web-search data is easily accessible and can be downloaded from Google Trends for free. We accessed Google Trends to generate a data pool of the search frequencies of the 90 selected neologisms as our source data. The search frequency data is critical to our model-fitting study as it is a reasonable approximation of how the words spread over the internet and the frequency of its use.

Although there are divergent views on the use of Google-hosted language big data for research in the humanities and language sciences [38], there are already several studies based on Google N-gram [39] and the Google N-gram viewer in the literature. These studies typically focus on longer term trends of lexical variations at the macro- [40] or micro-levels [6], or in a specific social context [41]. In addition, there are also demographic studies using internet usage data [42]. However, we believe that our study is the first study on language changes based on Google Trends.

### Data filtering

Although 咬文嚼字 *Yao Wen Jiao Zi* is one of the most authoritative periodicals on lexicography in Chinese, not all of the 90 neologisms proposed by the journal can be regarded as the “successful” neologisms in the sense of having entered the common lexicon. This is due partly to the selection criteria of being relevant for the year, which may not be long enough for some of the neologisms to fully develop (especially for those emerging in the latter half of the year). Hence, a number of words have been excluded from further analysis according to the following criteria:

**A**. **Data scarcity, including the following two sub-categories**:
  a) We noticed that some words did not have enough data provided by Google Trends to adequately test our model fitting. Seven neologisms were excluded due to this reason: 不抛弃不放 *bu4 pao1qi4 bu4 fang4qi4* “never cast away and never give up”; 不折腾 *bu4 zhe1teng0* “don’t bother”; 被就业 *bei4 jiu4ye4* “to be falsely categorized/certified as employed”; 打虎拍蝇 *da3hu3pai1ying2* “to beat a tiger and swat a fly (metaphorically refers to the fight against government corruption)”; 剁手党 *duo4shou3dang3* “shopaholic”; 获得感 *huo4de2gan3* “sense of acquisition”; and 互联网+ *hu4lian2wang3jia1* “internet+”.
  b) The patterns of some words showed only ascending trends, suggesting that they are still in the emergent stage and should be revisited later. Hence, five words were excluded: e.g., 断舍离 *duan4she3li2* “Danshari (KonMari) Method”; 颜值 *yan2zhi2* “attractiveness of appearance”; 网红 *wang3hong2* “internet celebrity”; 吃瓜群众 *chi1gua1qun2zhong4* “silent onlookers”; 葛优躺 *ge3you1tang3* “slouching on chair (like Ge You)”.
**B**. **Some words share the same word-form with other meanings/interpretations/functions**:
  Our analysis shows that for most of these neologisms, each word-form stands for multiple words or has multiple meanings, including established and conventionalized meanings and new interpretations [43]. As the Google Trends data is word-form based and not disambiguated, the actual frequency of the neologisms cannot be teased apart from the usages of the conventionalized meaning. In these cases, the pattern is a sum of tendencies of more than one-word meanings and cannot be used for our current study. This category of words mainly includes the following five sub-categories.
  a) We first excluded the words that did not acquire a new meaning, but simply had an old meaning that gained an increase popularity due to external incidents. Strictly speaking, these are not neologisms (which must involve either new meaning or new form). For example, 任性 *ren4xing4* “willful” was a mid-frequency word. Its usage and frequency surged when it started to be widely used in formal speeches in the conference of the People’s Congress and Political Association in 2015. Seven words, in addition to 任性 *ren4xing4* “willful”, were excluded based on this criterion: 钓鱼 *diao4yu2* “entrapment”, 套路 *tao4lu4* “tricks”, 小目标 *xiao3mu4biao1* “low-hanging fruit (ironic)”, 和 *he2* “peace”, 正能量 *zheng4neng2liang4* “positive energy”, 雷 *lei2* “thunder-struck”, 创客 *chuang4ke4* “entrepreneurs”.
  b) We also excluded the nouns that acquired a new function, such as addressing terms and/or pronominal functions. It is not clear if they acquired “new meaning”. For example, the word 宝宝 *bao3bao0* “baby” is a very common word in modern Chinese to address infants and later in life someone dear to the speaker. However, on the internet beginning in 2015, it acquired the new usage as a stance marker for self-reference, i.e., a special first person (and occasionally second person) pronoun. Based on this criterion, two words are excluded: 宝宝 *bao3bao0* “baby”, 亲 *qin1* “my dear”.
  c) We further excluded neologisms that retained the same meaning but extended their usages into different semantic domains as it is not clear if this meaning shift adds a new meaning or not. Two examples fall in this category: 顶层设计 *ding3ceng2she4ji4* “top-down design”, 神器 *shen2qi4* “artifact (gaming)”.
  d) We excluded neologisms that borrow an existing form but is used less frequently than the original form. As we mentioned above, since Google Trends provides word-form based usage data, it is not possible for the current study to differentiate the usages of neologisms. Based on this criterion, 光盘 *guang1pan2* “clean the plate or finish the food on your plate” was excluded as this expression was still predominantly used to refer to compact disc (i.e., CD), and only the compound form 光盘行动 *guang1pan2xing2dong4* “clean the plate/finish your food campaign” implies a new meaning.
  e) Another subset of neologisms (3 neologisms) sharing old forms but even more challenging to identify are those involving affixes or bound roots, such as 微 *wei1/wei2* “micro-”, 控 *kong4* “fervent fan” and 裸 *luo3* “naked, without strings attached”. These forms cannot stand alone but can productively form new words or compounds. These bound morphemes could become neologisms if they acquire new meanings without losing their word-formation potentials, e.g. 微 *wei1/wei2* “micro-”, and 裸 *luo3* “naked, without strings-attached”. A neologism can also be formed when a word acquires a new meaning and the productive word formation potentials at the same time, e.g. 控 *kong4* “fervent fan” has the original meaning of “to control”. Such forms pose multiple challenges to differentiate, either in text or in Google Trends. For instance, the criteria to identify bound and non-bound usages are complicated and highly contentious issues among linguists. In addition, the same compounds often retain both original and novel meanings. For instance, 裸婚 *luo3 hun1* could refer to the new meaning of “marriage without traditional ceremonies”, or the original meaning of “getting married naked”.

### Pattern of internet neologisms

**Fig 1:**
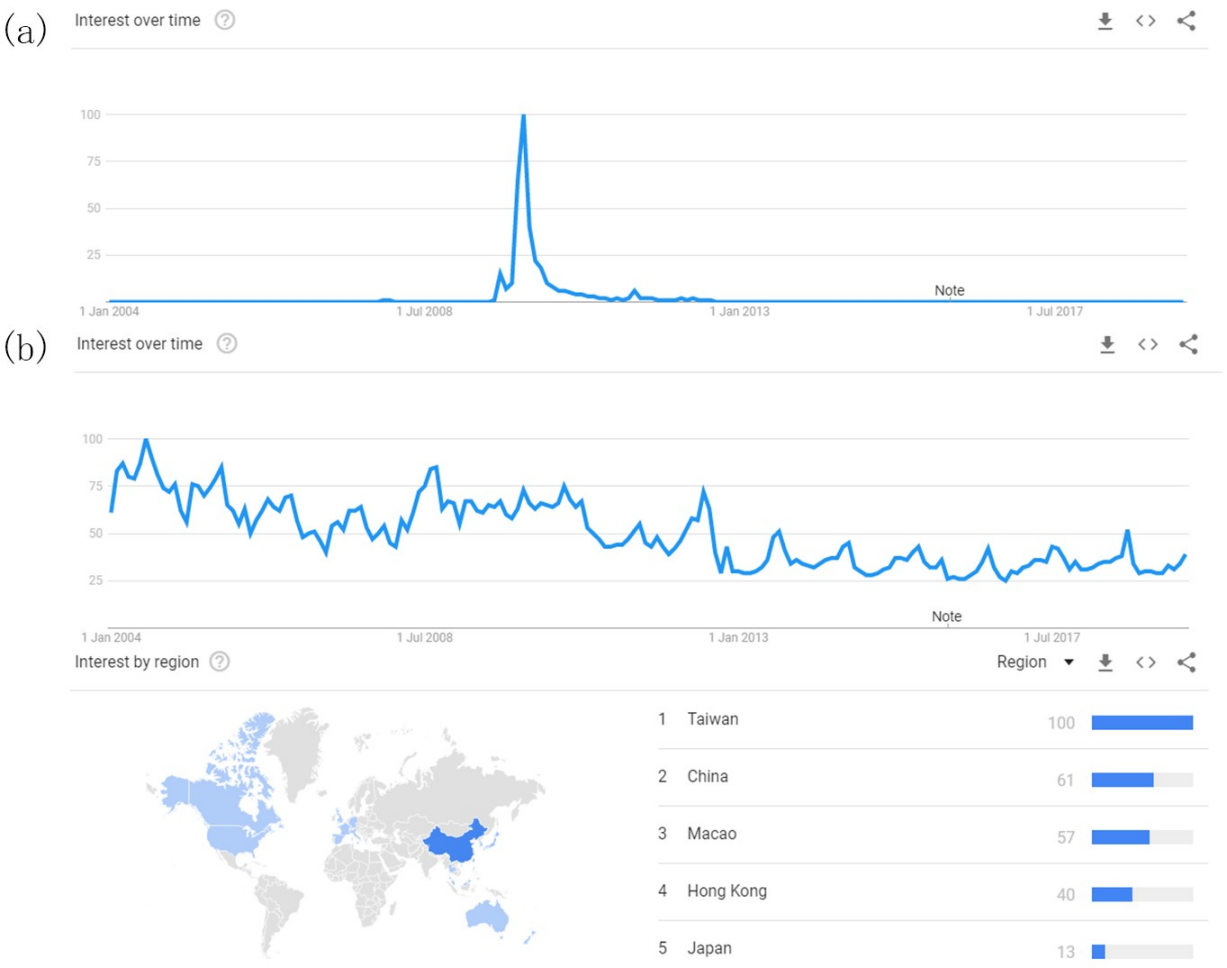
Interface of Google Trends. (a) The graph shows the search result of a sample neologism 蜗居 wo1ju1 “living within a snail’s shell” in Google Trends with a typical sharp rise and decay pattern. (b) The snapshot of the search result for the neologism 雷 lei2 “thunder, describing a person getting shocked by something absurd”. This word’s search frequency variation cannot be included in the paradigm of the sharp rise and decay pattern. The spatial distribution of the search frequency all over the world are also provided on the web page.

After excluding 28 neologisms based on the above criteria, 62 neologisms were included for model-fitting. In terms of our first research question, our initial analysis of the data from Google Trends shows that the popularity *P*(*t*) over time *t* of these 62 internet neologisms (see the wordlist in S1-S9 Tables in S1 File) have similar sharp rise and decay patterns (Fig 1(a)).

**Fig 2:**
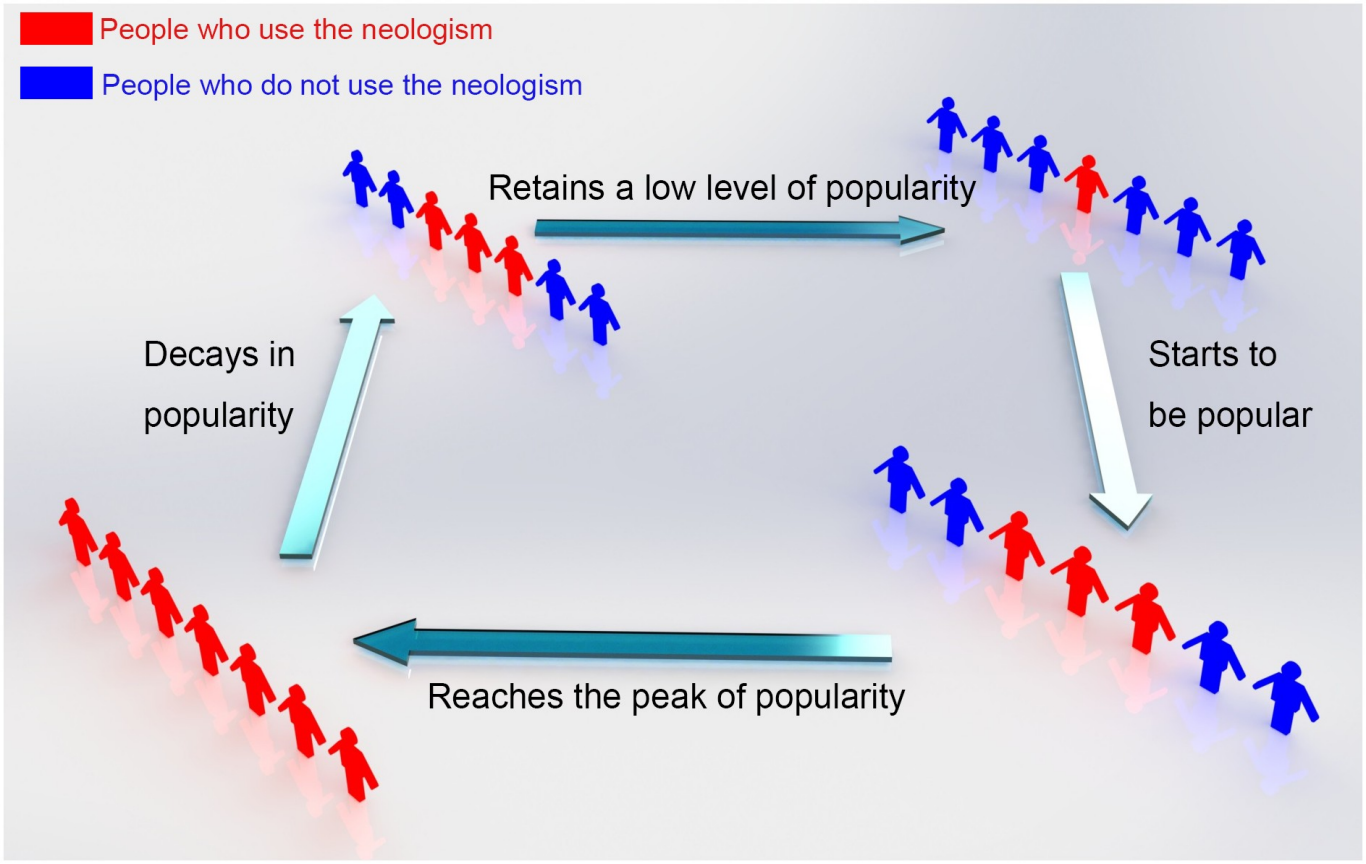
Four stages of neologisms’ lifetime. A schematic graph showing the four stages of an internet neologism’s lifetime, including a rise in popularity, a retaining of popularity, a process of decay in popularity, and a subsequent retention of a low level of popularity.

The prevalence of the 62 neologisms shows a clear rise-decay pattern with four stages. In the first stage, the neologism starts to be popular, and the search frequency increases. In the second stage, the word’s popularity reaches its peak at the normalized value of 100 and sustains that for a short while. The decaying begins in the third stage, as the popularity finally drops to a low value that is typical of a non-frequently used word. In the fourth stage, there is a sign that it is no longer popular, as shown in Fig 2.

### Data processing

The default range of the timescale is set from the year 2004 to the present. However, the usage index of some of the words around the peaks may jump from 0 to 100. When this occurred, we reduced the time span between sampling points to obtain more details for fitting purposes; i.e., we focus on the period when the neologism became popular. For instance, if a word was included in 咬文嚼字 *Yao3Wen1Jiao2Zi4* as a successful neologism in 2013, the range of the data’s timescale is set from January 1st of the preceding year (2012) to December 31st of the following year (2014).

**Fig 3:**
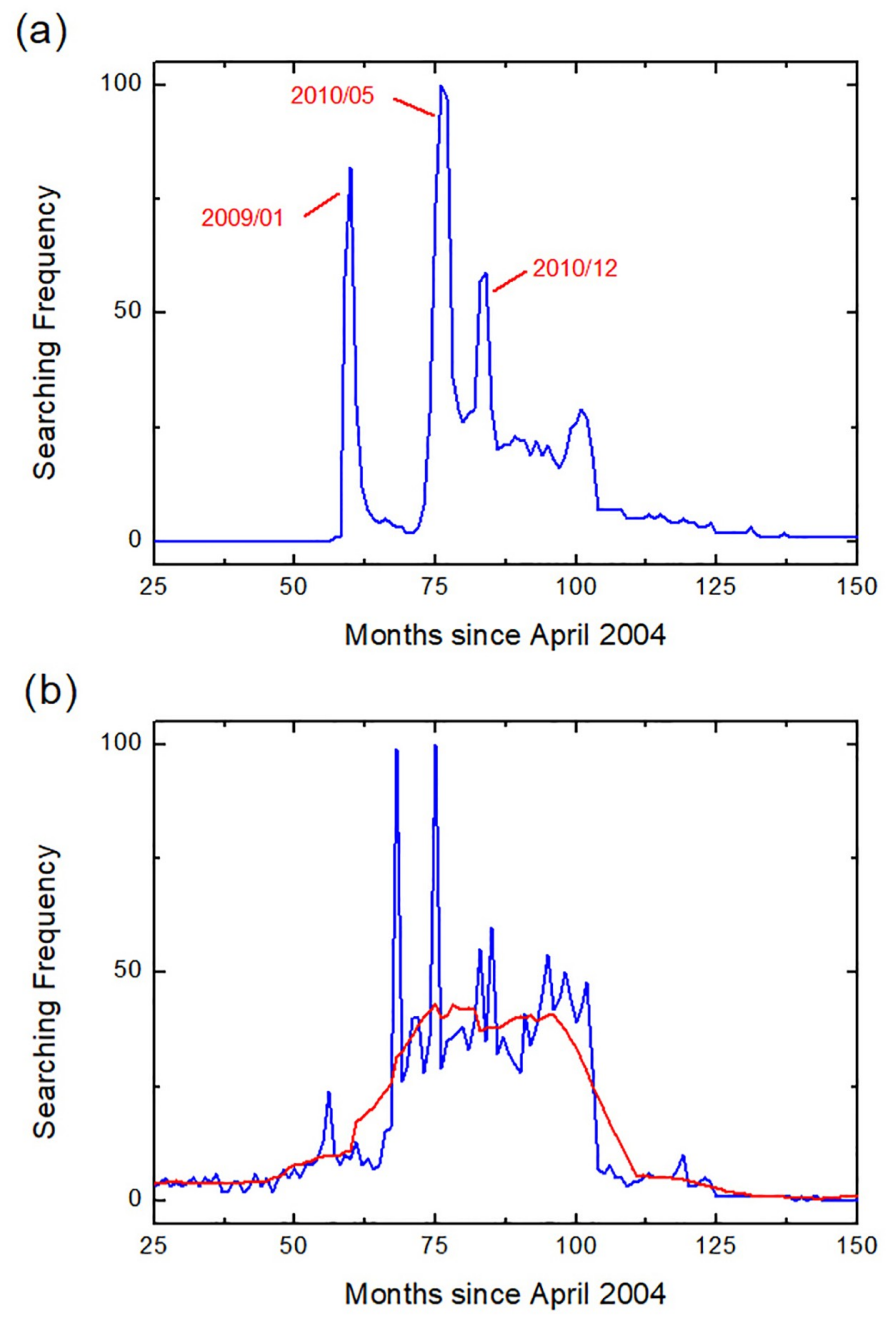
Data processing samples. (a) shows the data of the 2008 neologism 非诚勿扰 fei1cheng2 wu4rao3 “Serious Suitors Only” with multiple peaks at January of 2009, May of 2010, and December of 2010 respectively. (b) gives an example of data smoothing, where the raw data and the smoothed data are indicated by the color blue and red respectively. The smoothing process helped us to pick up the main evolution tendency of the word’s popularity by eliminating the disturbances caused by data noise.

There are multiple peaks for some neologisms such as 非诚勿扰 *fei1cheng2 wu4rao3* “Serious Suitors Only” (neologism in 2008), as shown in Fig 3(a). The multiple peaks may be triggered by relevant social events. For example, the release of a movie with the title 非诚勿扰 *fei1cheng2 wu4rao3* “Serious Suitors Only” may have caused the prevalence of this word in 2008. Then, the famous dating TV show with the same title and the sequel of the movie may trigger the next two peaks in 2010 and 2011 respectively. For such cases, we select the peak of the year when the neologism first became popular (indicated by the journal 咬文嚼字 *Yao3Wen1Jiao2Zi4*) for modeling purposes.

It is noted that even though the rising and the decaying tendency can be observed, there are fluctuations (or noise) in some data sets (such as 秒杀 *miao3sha1* “seckill” as shown in Fig 3(b). To address such problems, we performed the data smoothing operation using the data processing software (Origin) to smooth the curve for fitting purposes.

The fluctuating pattern of 秒杀 *miao3sha1* “seckill” can be explained by its high correlation with online sales events. Any big traditional sales dates (such as November 11th: the biggest sales date on TaoBao, China’s biggest e-commerce website) may bring large spikes, and therefore cause the fluctuations and noises in its overall tendency. The example demonstrates that carefully chosen words can offer good evidence for collective human behavior. In fact, 秒杀 *miao3sha1* “seckill” showed clear spikes around big dates for online commerce activities, such as 11/11. Hence, it can be used to corroborate and/or discover patterns of commercial activities.

## Results

### Model fitting

The homothetic *P*(*t*) pattern of the majority of internet neologisms is very similar to the real life cycles of living organisms, which suggests a correlation between language development and biological evolution [44, 45]. Given that the life cycle of neologisms involves the spread of words among the human population, we have postulated to model it as an epidemic model.

When comparing the spread of words to the model of epidemics, there are several similarities between the spreading of neologisms and the spreading of diseases that can be observed: a sudden spike in infections/usage, infectious diffusion among the population/speakers, and a short lifetime after the peak. For the current study, we adopt a simple and powerful computational model of epidemiology to represent the observed *P*(*t*) of neologisms. The SIR (Susceptible Infectious Recovered) model is a host (population) based model as it takes into account three variables that are directly dependent on the population: the susceptible, the infected, and the recovered individuals [46]. The SIR model interprets the development of a certain virus in terms of predictions related to the numbers of infected persons at arbitrary time nodes. It is also reliable in predicting the peak period for the infection.

The model assumes there are three classes of people: susceptible ones, infectious ones, and recovered ones. All of these are functions of time *t*, and can be written as *S*(*t*), *I*(*t*) and *R*(*t*). Thus, the differential equations to describe the virus transmission are expressed as:

$$\begin{array}{c} \left\{ \begin{array}{c} \frac{dS(t)}{dt}=-\beta I\left(t\right)S\left(t\right),\\ \frac{dI(t)}{dt}=\beta I\left(t\right)S\left(t\right)-\alpha I\left(t\right),\\ \frac{dR(t)}{dt}=\alpha I\left(t\right), \end{array}\right. \end{array}$$ (1)

where *α* and *β* are parameters to characterize the increasing rates of recovery and infectious people respectively. It should be noted that the equations above have no analytical solutions. However, we can estimate the *α* and the *β* by fitting the *I*(*t*) derived from Eq (1) with the real popularity of a given neologism obtained from Google Trends numerically. Essentially, the SIR model involves competition between the hosts’ immunity *α* and the level of infectivity of the virus *β* leading to an inflection point where the increasing number of recoveries starts to be bigger than the number of infected people and the evolution pattern of the neologism turns from the rising stage to the decaying stage.

**Fig 4:**
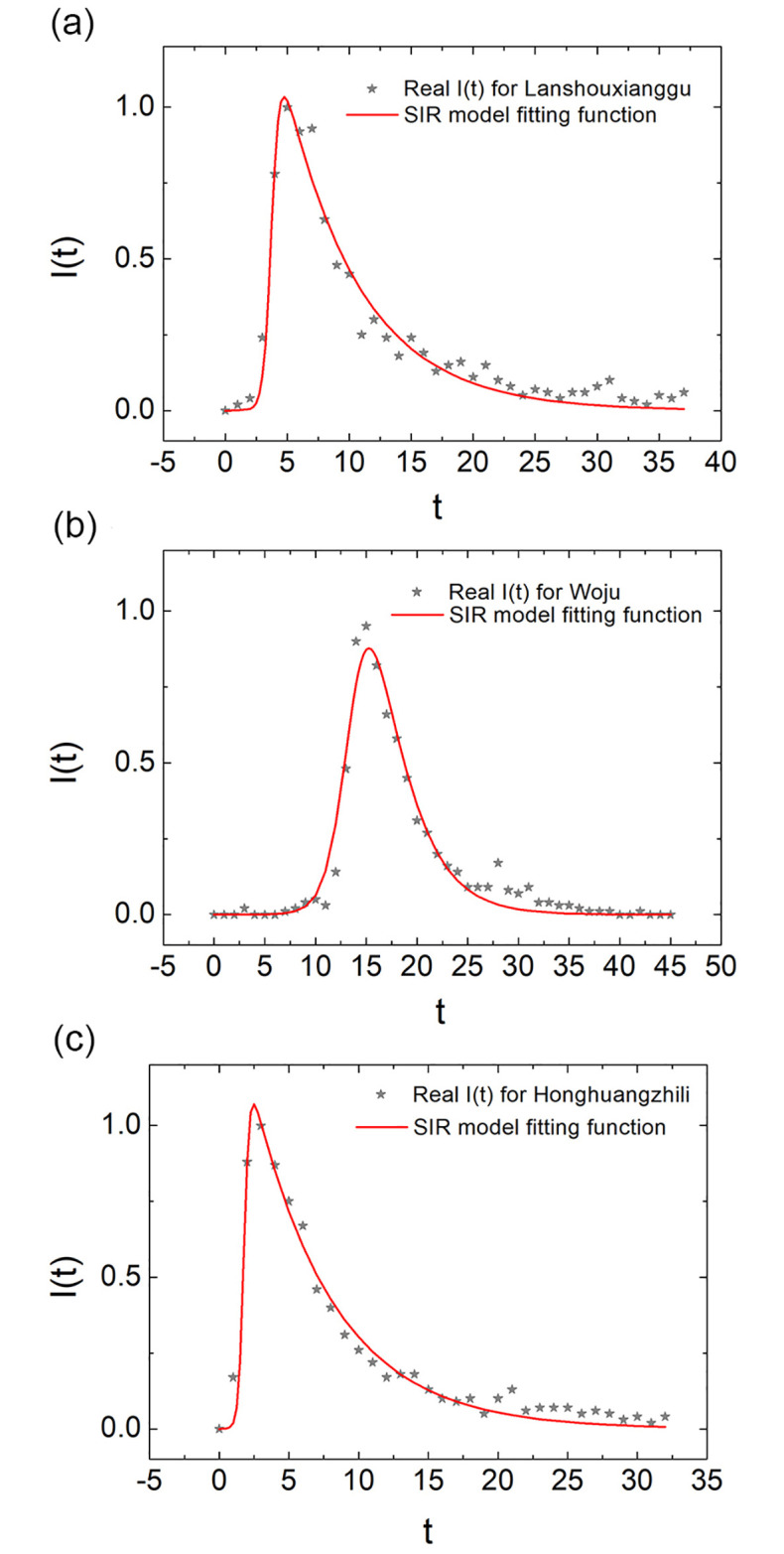
The performance of the model fitting. The SIR model fitting results are as illustrated. In the picture, the *P*(*t*) data obtained from Google Trends are grey stars. The SIR model fitting functions are denoted by the red lines. (a),(b),(c) gives the data of 蓝瘦香菇 lan2shou4xiang1gu1 “too sad to cry”, 蜗居 wo1ju1 “living within a snail’s shell/small room”, 洪荒之力 hong2huang1zhi1li4 “the force from primitive period” respectively, as well as the SIR fitting functions.

Henceforth, we acquire the *P*(*t*) data from Google Trends and fit them with the fitting functions given in Eq (1) by setting *I*(*t*) = *P*(*t*). In Fig 4, we illustrate the fitting results of three neologisms with a typical rise and decay pattern. To estimate the fitting effects, we adopt the fitting parameter $R^{2}\equiv 1-\Sigma {\left(f_{i}-x_{i}\right)}^{2}/\Sigma {\left(\bar{x}-x_{i}\right)}^{2}$. In the expression, *x*_*i*_, *f*_*i*_ are the i^th^ values of the data set and fitting function respectively, and $\bar{x}$ is the average value of the whole data. If *R*^2^ → 1, the fitting function is in good agreement with the actual data. We then ran the statistics of all of the neologisms with the rise and decay pattern and obtained a mean value *R*^2^ = 0.8975 for the SIR (epidemic) model (See S10 Table in S1 File for more details).

### Summary of model fitting results

To answer the second research question, the fitting parameter *R*^2^ of the SIR epidemiological model indicates that mathematically the model successfully describes the neologisms’ life cycles.

Recall that we mentioned that the epidemic model is an *a priori* model in the Introduction. The epidemic model is derived from the competition between the recovery rate *α* and the infection rate *β* and the position of the inflection point where the number of recoveries exceeds the number of infections associated with the ratio *β*/*α*. This allows the epidemic model to predict the inflection point, unlike the memetic model, which does not inherently indicate the emergence of an inflection point.

**Fig 5:**
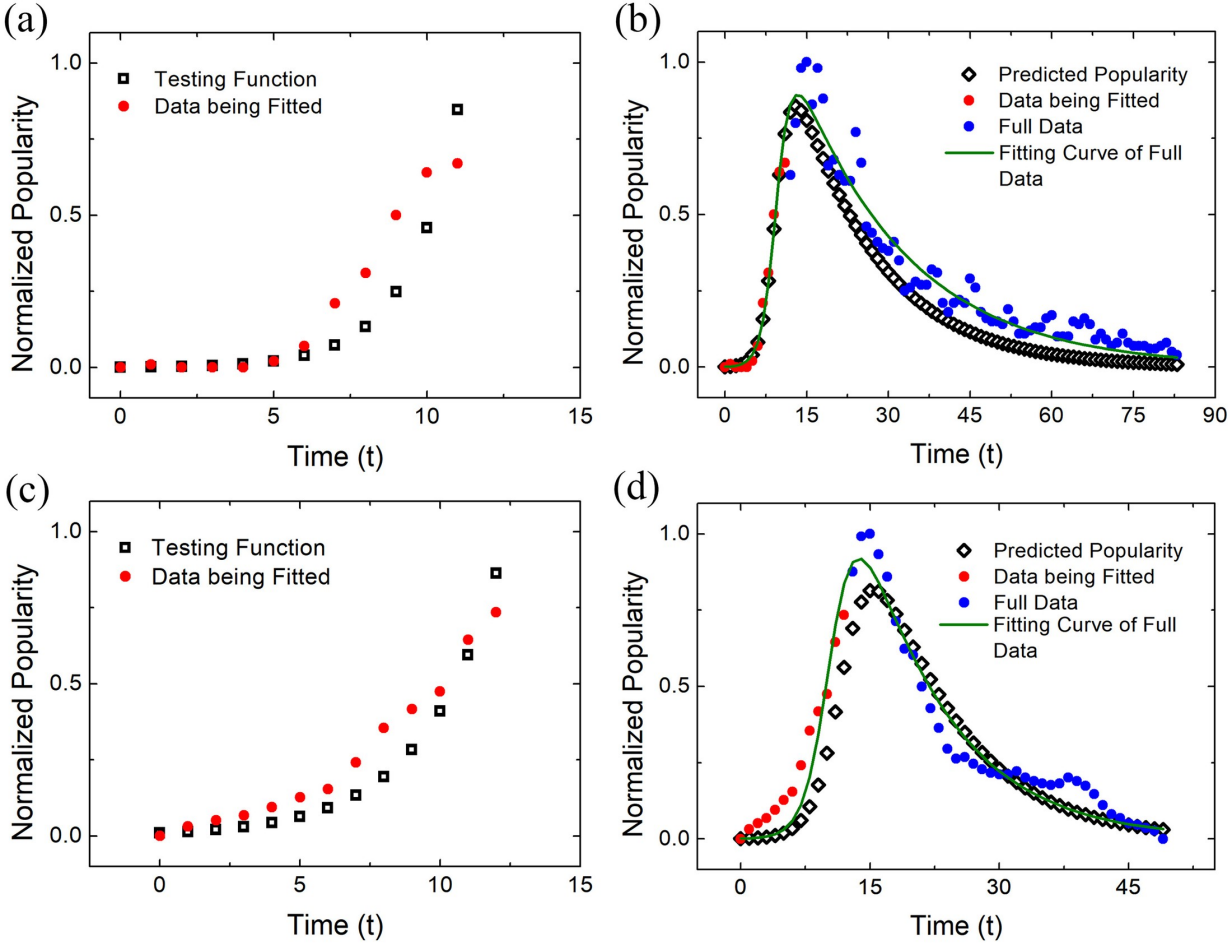
The predictive power of the SIR model. One can use the SIR model to predict the popularity evolution of a neologism based on a small amount of data before reaching the inflection point. A fraction of data to be fitted and the remaining data are denoted by red and blue circles respectively. In order to derive *α* and *β*, the testing exponential functions denoted by black squares are used to fit with the data of 舌尖上 she2jian1shang4 “on the tip of the tongue” and 低碳 di1tan4 “low-carbon” as shown in (a) and (c) respectively. By utilizing the parameters derived from this step, we can obtain the predicted popularity evolution over time, indicated by the black diamonds, according to the small amount of data, which is very close to the green fitting curves yielded by fitting the full data, as illustrated in (b), (d).

Two examples are given in Fig 5 to illustrate the predictive power of the SIR model. As the number of initial patients *I*(0) is very small compared to the total number susceptible people, the rising part of the SIR model can be analytically described by the function *I*(*t*) = *I*(0)*Exp*[(*β* − *α*)*t*]. By fitting with this testing function we can determine the values of *α* and *β* with a small amount of data before reaching the inflection point as shown in Fig 5(a) and 5(c), in which small fractions of data of 舌尖上 *she2jian1shang4* “on the tip of the tongue” and 低碳 *di1tan4* “low-carbon” denoted by red circles are selected to be fitted. The testing exponential function is indicated by black squares.

After obtaining the *α* and *β*, we can use the differential equations of the SIR model to predict the complete evolution pattern based on a small part of the data at the early rising stage. As demonstrated in Fig 5(b) and 5(d), based on the selected data, denoted by red circles, we can derive the predicted popularity over time, which is indicated by black diamonds. For comparison, the SIR fitting curve derived from the full data denoted by the color green is also given. We can see that the predicted popularity evolution based on a small fraction of data is very close to the fitting result based on the full data, which indicates that the epidemic model has the ability to predict the location of the inflection point.

## Discussion

We have demonstrated that the propagation and life cycle of neologisms can be *a priori* described by a host-driven SIR epidemic model. Our model fitting study demonstrated that the SIR model has a good fitting effect for describing the trajectory of the neologisms and can make good predictions of the location of the peak inflection point. Thus, the SIR model allowed us to gain insight into the epidemic-like spreading patterns behind the prevalence of internet neologisms.

The SIR (epidemic) model is known to be a dynamic model. The dynamic nature of the model follows from the fact that the numbers S, I, and R represent subsets of potential hosts, which is fixed in total. In addition, these subsets have both competition and co-development relations among them. In either scenario, the changes of the variables are in fact the result of interaction of the sentient hosts with the non-sentient spreading of words/virus. In other words, when modeling events as the result of the interaction between sentient and non-sentient actors, it is possible to implicitly model the impact of the non-sentient actors by tracking variations of the sentient actors alone, but it is much harder (if not impossible) to do so in the other direction.

What the SIR model tries to model is the dynamic relation among S (Susceptible), I (Infectious), and R (Recovered). For an epidemic to spread widely, in general the susceptible and infected population should be maximized; which means that the recovered population should be relatively small (or at least would take longer to recover). However, if the recovered population is minimized, it means that fewer of the infected people have recovered. This, in turn, quickly decimates the infected population. Yet a very small infected population means that the susceptible population is also proportionally smaller as there are fewer to spread the disease. In sum, for an epidemic to spread effectively, it requires that the pathogen, such as a virus, achieves some degree of symbiosis with the host (the human). To maximize the life-span as well as the spread of the epidemic, the pathogen needs to effectively infect the susceptible individuals, and to last long enough on the host without killing him/her to allow for effective propagation.

Similarly, for a new word to be spread, the host (i.e., the person who reads/hears the new word) must accept and integrate it in his/her mental lexicon in order to use it again (and to use it frequently). Moreover, the infected (those who encountered the new word) must be ready to pass it on to the others (the susceptible individuals). If the infected individual is not ready to use the new word, then the individual “recovers” and is no longer a host in the life cycle of the neologism. In this case, it is not the robustness of the host (the speaker), but the robustness of the new word that is more important. If a new word is heard but not used, or used but receives no attention, then it cannot spread effectively and therefore will have a short life cycle. That is, whether the neologism can enter and “live” in the speaker’s mental lexicon is similar to whether a pathogen can achieve a degree of symbiosis with the host. In other words, a long-life cycle (i.e., being “successful”) for an epidemic/neologism requires symbiosis between the virus and the host (for an epidemic) as well as sustainable inclusion in the mental lexicon of the host (for a neologism).

Given that the life cycle of a neologism is the result of dynamic human interaction, we could try to interpret our modeling results with the S-curve frequency development predicted for most complex systems involving cognitive behaviors [47–49]. Note that the S-curve model of complex systems expect a change to start slowly, followed by a period of rapid development, and then a plateau with possible residues when the change is completed. The two relative slow periods of change are the two legs of the S, and the rapid rise (or fall) is often described as the snowball effect [50, 51]. The epidemic model should be able to model the sharp rising snowball effect portion of the life cycle.

However, note that one salient discrepancy between the S-curve model and our neologism data based on internet searches is the sharp decay soon after reaching a stable stage (i.e., being conventionalized), instead of a leveling-off stage described in the S-curve. We suspect that there are two possible reasons for this discrepancy. First, the literature [51] shows that the S-curve model best describes the replacement changes where a new form replaces an old form, and only when the replicator selection condition is met. Most of the neologisms that we studied do not belong to this category, including the neologisms with the potential to lead to a replacement change. A good example is 围脖 *wei2bo2* “scarf/micro-blog”, which competes with its near-homophone 微博 *wei1bo2/wei2bo2* “micro-blog”, and settled as a tongue-in-cheek euphemism, but did not serve as a replacement term. This is probably due to the strong daily usage meaning of scarf. Second, it may be attributed to the difference between search behavior and language use behavior. That is, once a word is conventionalized and speakers have certain familiarity with the word, there is no need for “dictionary look up” queries to determine the meaning of the words. To the best of our knowledge, there is no way to differentiate “word-meaning search” from “content search” but it is reasonable to assume that a drastic drop of “word-meaning search” could contribute to the decay of frequency of the internet usage data after it has reached its peak.

To test the robustness of our findings, in the future we could extract the most popular neologisms that eventually did not successfully enter the lexicon. By fitting these presumably less successful neologisms with the epidemiology model, we can test whether this model can also predict unsuccessful neologisms in spite of their high frequency of use. This result would complement our current result and help us to explicate more completely the features contributing to the life cycle of neologisms. Another issue for future research is the role of the linguistic features of neologisms in determining its vitality. For instance, previous studies in English often showed that word-length (in terms of the number of syllables) plays a role in the lifespan of a neologism [15]. However, it is well-established that the majority of Chinese words (both in terms of type and token) are either mono- or di-syllabic [52]. In addition, mono-syllabic words have higher token frequency, but bi-syllabic ones are ahead in type frequency [52, 53]. Other features, such as tonal motif or rime patterns [54] may also be explored.

## Conclusion

In this paper, we adopted internet-based data from Google Trends for the temporally marked popularity of neologisms in Chinese in order to model their propagation and life cycle. We argued that the memetic model was not suitable for modeling the fast evolution of internet neologisms and proposed, instead, to model the collected data by a host-driven epidemic model. We found the SIR model, a host-driven dynamic computational model for epidemiology, provided not only a good description of the life cycle of the neologism, but also a powerful predictive ability to locate the inflection points. Our study provides a new method to obtain statistical data for neologisms from an internet search engine, and proposes a classical mathematical model used for epidemics to describe the life cycle of neologisms. This model indicates that the propagation of words is similar to the propagation of diseases. Moreover, our results also suggest that a host-driven dynamic model is well fitted to model cognitive behaviors for a large population.

Previous research showed that language evolution data and modeling can be used to support models of genetic and biological evolution [44, 55, 56]. Our paper supports this line of modeling as we have shown that the life cycle of neologisms fits well within an epidemic model. Another new research approach has been recently proposed in [57] to correlate significant typological variations of weather words to meteorological patterns. The basic hypothesis is that the environmental impact, as measured by kinesis, would be reflected in the linguistic system of weather expressions [58]. Future work may investigate if it is possible to model the development of an epidemic by measuring the use of epidemic-related words, including not only neologisms but also words that are conceptually related to the epidemic such as symptoms, methods for prevention, as well as treatment. This will be crucial in at least two contexts: either at the early stage of an emergent epidemic when there is not enough clinical data (or when the clinical data is not released for whatever reason). The second context has to do with historical epidemics, such as in the history of China, where there is no clinical data. Since China had many local gazetteers in different cities (地方志 *di4fang1zhi4* “local gazetteers”), modeling the cycle of changes of symptomatic, preventative, or medicinal terms based on historical data of local gazetteers over a period of time and from different places in the same region, it may be possible to establish the model of the spread of major epidemics in a historical context. Either research direction can be enriched by correlational analysis with environmental changes, such as major weather events, based on language data analysis [57].

Last, but not least, our study has shown that, with regard to the spread of novel ideas involving internet-based language mediated behaviors, an epidemic model is well suited to model human behavior. We have mentioned above that the epidemic model is dependent on data obtained as the result of the actions of hosts. Thus, the role of hosts, i.e., human beings, and their complex interactions are included as potential variables. This is different from memetic models which are based on the assumption that memes are selfish and hungry [21, 22] in the sense that their only goal is to maximize their growth and life-span. Interestingly, the standard epidemic model of modeling susceptible, infectious, and recovered persons in fact allows for altruistic behavior and not just the natural drive for survival of the fittest. That is, by inclusion of the “recovered” as a variable, it allows the society to collaborate in some way, and not just act as the sum of competing individuals, as predicted by other models, such as the memetic model. In this way, the epidemic model predicts the life cycle of neologisms with novel forms and/or meaning because their growth and spread require collaborative efforts from the hosts as they are not near-facsimiles of existing words, and thus, cannot rely on frequency alone to propagate. This interpretation crucially implies that language data provides potentially critical empirical evidence for collective human behavior changes and should be explored as a fertile ground for future research.

## Supporting information

S1 File (PDF)
