# Supplementary material for: Neologisms are epidemic: Modeling the life cycle of neologisms in China 2008-2016
Source: PLoS One. 2021 Feb 3;16(2):e0245984. doi: 10.1371/journal.pone.0245984 (PMC7857598; doi:10.1371/journal.pone.0245984)
Supplement: S1 File — (PDF) [file pone.0245984.s001.pdf]

Meng Han Jiang[1,2¶], Xiang Ying Shen[3,7], Kathleen Ahrens[4,6] Chu Ren Huang[2,5*]

1 Department of Chinese Language and Literature, Peking University, Beijing, China
2 Department of Chinese and Bilingual Studies, The Hong Kong Polytechnic University, Hong Kong, China
3 Department of Physics, Chinese University of Hong Kong, Hong Kong, China
4 Department of English, The Hong Kong Polytechnic University, Hong Kong, China
5 HK PolyU-PKU Research Centre on Chinese Linguistics, Hong Kong, China
6 Research Centre for Professional Communication in English, The Hong Kong Polytechnic University, Hong Kong, China
7 Shenzhen JL Computational Science And Applied Research Institute, Shenzhen, China

¶menghan.jiang@connect.polyu.hk
* churen.huang@polyu.edu.hk

Tables

Table S 1. Chinese Internet Neologisms for 2008

Word	Sharp rise and decay	Category for exceptions
山寨 *shan1zhai4* ‘reengineered copy’	Yes	
口红效应 *kou3hong2 xiao4ying4* ‘lipstick effect’	Yes	
拐点 *guai3dian3* ‘crossover point’	Yes	
宅男宅女 *zhai2nan2 zhai2nv3* ‘people who are housebound as a lifestyle choice’	Yes	
非诚勿扰 *fei1cheng2 wu4rao3* ‘Serious Suitors Only (the most-viewed dating show in China)’	Yes	
囧 *jiong3* ‘embarrassment’	Yes	
雷 *lei2* ‘thunder-struck’	No	Multiple meanings (a)
和 *he2* ‘peace’	No	Multiple meanings (a)
不抛弃不放弃 *bu4 pao4qi4 bu4 fang4qi4* ‘never cast away and never give up’	No	Data scarcity (a)
不折腾 *bu4 zhe1teng0* ‘don’t bother’	No	Data scarcity (a)

Table S 2. Chinese Internet Neologisms for 2009

Word	Sharp rise and decay	Category for exceptions
不差钱 *bu4 cha4qian2* ‘rich’	Yes	
躲猫猫 *duo3 mao1mao1* ‘hide and seek’	Yes	
低碳 *di1tan4* ‘low-carbon’	Yes	
纠结 *jiu1jie2* ‘be entangled with’	Yes	
秒杀 *miao3sha1* ‘seckill’	Yes	
蜗居 *wo1ju1* ‘living in a tight space’	Yes	
蚁族 *yi3zu2* ‘ant tribe’	Yes	
被就业 *bei4jiu4ye4* ‘to be falsely categorized/certified as employed’	No	Data scarcity (a)
裸 *luo3* ‘naked, without string-attached’	No	Multiple meanings (e)
钓鱼 *diao4yu2* ‘entrapment’	No	Multiple meanings (a)

Table S 3. Chinese Internet Neologisms for 2010

Word	Sharp rise and decay	Category for exceptions
给力 *gei3li4* ‘awesome’	Yes	
神马都是浮云 *shen2ma3 dou4shi4 fu2yun2* ‘all is vanity’	Yes	
围脖 *wei2bo2* ‘scarf/micro-blog’	Yes	
围观 *wei2guan1* ‘gathering of onlookers’	Yes	
二代 *er4dai4* ‘scion’	Yes	
拼爹 *pin1die1* ‘riding on Dad’s coattail’	Yes	
表情帝 *biao3qing2di4* ‘walking emoji’	Yes	
达人 *da2ren2* ‘person who is good at a specific task’	Yes	
穿越 *chuan1yue4* ‘travel back in time’	Yes	
控 *kong4* ‘be addicted to’	No	Multiple meanings (e)

Table S 4. Chinese Internet Neologisms for 2011

Word	Sharp rise and decay	Category for exceptions
伤不起 *shang1 bu4 qi3* ‘fragile’	Yes	
hold住 *hold zhu4* ‘things are under control’	Yes	
我反正信了 *wo3 fan3zheng4 xin4le0* ‘I choose to believe it (ironical use)’	Yes	
坑爹 *keng1die1* ‘you (could have) fooled me’	Yes	
卖萌 *mai4meng2* ‘acting cute’	Yes	
吐槽 *tu3cao2* ‘to embarrass by spoiling beans’	Yes	
气场 *qi4chang3* ‘charisma’	Yes	
悲催 *bei1cui1* ‘tragic’	Yes	
忐忑 *tan3te4* ‘be mentally disturbed’	Yes	
亲 *qin1* ‘my dear’	No	Multiple meanings (b)

Table S 5. Chinese Internet Neologisms for 2012

Word	Sharp rise and decay	Category for exceptions
元芳你怎么看 *yuan2fang1 ni3 zen3me0 kan4* 'Yuanfang, what do you think?'	Yes	
舌尖上 *she2jian1 shang4* 'on the tip of the tongue, gustatory'	Yes	
躺着也中枪 *tang3zhe0 ye3 zhong4qiang1* 'blindsided innocently'	Yes	
高富帅 *gao1shuai4fu4* 'a man who is tall, rich and handsome'	Yes	
中国式 *zhong1guo1 shi4* 'Chinese style (ironic)'	Yes	
压力山大 *ya1li4shan1da4* 'the pressure is as heavy as the mountain'	Yes	
赞 *zan4* 'praise'	Yes	
最美 *zui4mei3* 'the best'	Yes	
接地气 *jie1di4qi4* 'grounded'	Yes	
正能量 *zheng4neng2liang4* 'positive energy'	No	Multiple meanings (a)

Table S 6. Chinese Internet Neologisms for 2013

Word	Sharp rise and decay	Category for exceptions
中国梦 *zhong1guo2 meng4* ‘the Chinese dream’	Yes	
倒逼 *dao4bi1* ‘backfire’	Yes	
逆袭 *ni4xi2* ‘counterattack’	Yes	
女汉子 *nv3 han4zi0* ‘tomboy’	Yes	
土豪 *tu3hao2* ‘tuhao, rich man’	Yes	
点赞 *dian3zan4* ‘give a thumbs-up’	Yes	
大V *da4V* ‘social media influencer’	Yes	
奇葩 *qi2pa1* ‘oddball’	Yes	
光盘 *guang1pan2* ‘clean the plate’	No	Multiple meanings (d)
微 *wei1/wei2* ‘micro-’	No	Multiple meanings (e)

Table S 7. Chinese Internet Neologisms for 2014

Word	Sharp rise and decay	Category for exceptions
新常态 *xin 1chang2tai4* 'the new norm'	Yes	
断崖 *duan4ya2* 'sharp drop off'	Yes	
你懂的 *ni3dong3de0* 'you know (and I don't have to say it)'	Yes	
失联 *shi1lian2* 'lost contact'	Yes	
高大上 *gao1da4shang4* 'high level'	Yes	
萌萌哒 *meng2meng2da1* 'so cute'	Yes	
断舍离 *duan4she3li2* 'Danshari (KonMari) Method'	No	Data scarcity (b)
神器 *shen2qi4* 'artifact (gaming)'	No	Multiple meanings (c)
顶层设计 *ding3ceng2she4ji4* 'top-down design'	No	Multiple meanings (c)
打虎拍蝇 *da3hu3pai1ying2* 'to beat a tiger and swat a fly (metaphorically refers to the fight against government corruption)'	No	Data scarcity (a)

Table S 8. Chinese Internet Neologisms for 2015

Word	Sharp rise and decay	Category for exceptions
脑洞大开 *nao3dong4da4kai1* ‘greatly enrich one’s mind’	Yes	
主要看气质 *zhu3yao4kan4 qi4zhi4* ‘beauty in confidence’	Yes	
剁手党 *duo4shou3dang3* ‘shopaholic’	No	Data scarcity (a)
获得感 *huo4de2gan3* ‘sense of acquisition’	No	Data scarcity (a)
互联网+ *hu4lian2wang3jia1* ‘internet+’	No	Data scarcity (a)
颜值 *yan2zhi2* ‘attractiveness of appearance’	No	Data scarcity (b)
网红 *wang3hong2* ‘internet celebrity’	No	Data scarcity (b)
宝宝 *bao3bao0* ‘baby’	No	Multiple meanings (b)
创客 *chuang4ke4* ‘entrepreneurs’	No	Multiple meanings (a)
任性 *ren4xing4* ‘has one’s own way’	No	Multiple meanings (a)

Table S 9. Chinese Internet Neologisms for 2016

Word	Sharp rise and decay	Category for exceptions
供给侧 *gong4ji3ce4* ‘supply side’	Yes	
工匠精神 *gong1jiang4 jing1shen2* ‘craftsmanship’	Yes	
洪荒之力 *hong2huang1zhi1li4* ‘with all one’s might’	Yes	
友谊的小船 *you2yi2 de0 xiao3chuan2* ‘friendSHIP’	Yes	
一言不合就 *yi4yan2bu4he2 jiu4* ‘to do X on a whim’	Yes	
蓝瘦香菇 *lan2shou4xiang1gu1* ‘sad, crying feeling’	Yes	
吃瓜群众 *chi1gua1qun2zhong4* ‘silent onlookers’	No	Data scarcity (b)
葛优躺 *ge3you1tang3* ‘slouching on chair (like Ge You)’	No	Data scarcity (b)
套路 *tao4lu4* ‘tricks’	No	Multiple meanings (a)
小目标 *xiao3 mu4biao1* ‘low-hanging fruit (ironic)’	No	Multiple meanings (a)

R^2 for neologisms

Table S 10. **R^2 for Chinese neologisms**

Word	R^2 for epidemic model
达人 *da2ren2* ‘person who is good at a specific task’	0.848594228
纠结 *jiu1jie2* ‘be entangled with’	0.850379462
新常态 *xin 1chang2tai4* ‘the new norm’	0.839228736
你懂的 *ni3dong3de0* ‘you know (and I don’t have to say it)’	0.892478409
萌萌哒 *meng2meng2da1* ‘so cute’	0.891936859
坑爹 *keng1die1* ‘you (could have) fooled me’	0.902316027
围脖 *wei2bo2* ‘scarf/micro-blog’	0.869607805
女汉子 *nv3 han4zi0* ‘tomboy’	0.86088291
一言不合就 *yi4yan2bu4he2 jiu4* ‘to do X on a whim’	0.771061234
接地气 *jie1di4qi4* ‘grounded’	0.813599959
逆袭 *ni4xi2* ‘counterattack’	0.811797854
忐忑 *tan3te4* ‘be mentally disturbed’	0.911037086
不差钱 *bu4 cha4qian2* ‘rich’	0.898707344
供给侧 *gong4ji3ce4* ‘supply side’	0.760419543
脑洞大开 *nao3dong4da4kai1* ‘greatly enrich one’s mind’	0.77977761
躲猫猫 *duo3 mao1mao1* ‘hide and seek’	0.934306947
我反正信了 *wo3 fan3zheng4 xin4le0* ‘I choose to believe it (ironical use)’	0.91795451
最美 *zui4mei3* ‘the best’	0.91585733
吐槽 *tu3cao2* ‘to embarrass by spoiling beans’	0.881055795
山寨 *shan1zhai4* ‘reengineered copy’	0.924371323
赞 *zan4* ‘praise’	0.872654439
伤不起 *shang1 bu4 qi3* ‘fragile’	0.893897962
拼爹 *pin1die1* ‘riding on Dad’s coattail’	0.880699785
大V *da4V* ‘social media influencer’	0.857058651
悲催 *bei1cui1* ‘tragic’	0.920461614
中国梦 *zhong1guo2 meng4* ‘the Chinese dream’	0.897254922
宅男宅女 *zhai2nan2 zhai2nv3* ‘people who are housebound as a lifestyle choice’	0.883684462
囧 *jiong3* ‘embarrassment’	0.903338783
低碳 *di1tan4* ‘low-carbon’	0.94267191
元芳你怎么看 *yuan2fang1 ni3 zen3me0 kan4* ‘Yuanfang, what do you think?’	0.959946193
高大上 *gao1da4shang4* ‘high level’	0.902310036
气场 *qi4chang3* ‘charisma’	0.861948563
友谊的小船 *you2yi2 de0 xiao3chuan2* ‘friendSHIP’	0.861895662
失联 *shi1lian2* ‘lost contact’	0.981707522
躺着也中枪 *tang3zhe0 ye3 zhong4qiang1* ‘blindsided innocently’	0.832880143
工匠精神 *gong1jiang4 jing1shen2* ‘craftsmanship’	0.778069443
中国式 *zhong1guo1 shi4* ‘Chinese style (ironic)’	0.922914639
卖萌 *mai4meng2* ‘acting cute’	0.91040885
舌尖上 *she2jian1 shang4* ‘on the tip of the tongue, gustatory’	0.943159984
蓝瘦香菇 *lan2shou4xiang1gu1* ‘sad, crying feeling’	0.957755233
给力 *gei3li4* ‘awesome’	0.961686029
高富帅 *gao1shuai4fu4* ‘a man who is tall, rich and handsome’	0.927065502
蜗居 *wo1ju1* ‘living in a tight space’	0.960013914
主要看气质 *zhu3yao4kan4 qi4zhi4* ‘beauty in confidence’	0.994989483

Table S 11. R^2 **for Chinese neologisms**

拐点 *guai3dian3* 'crossover point'	0.867517358
穿越 *chuan1yue4* 'travel back in time'	0.9628525
压力山大 *ya1li4shan1da4* 'the pressure is as heavy as the mountain'	0.868422111
hold住 *hold zhu4* 'things are under control'	0.892732207
围观 *wei2guan1* 'gathering of onlookers'	0.92493998
蚁族 *yi3zu2* 'ant tribe'	0.934640923
点赞 *dian3zan4* 'give a thumbs-up'	0.934085266
口红效应 *kou3hong2 xiao4ying4* 'lipstick effect'	0.89429118
秒杀 *miao3sha1* 'seckill'	0.87614209
洪荒之力 *hong2huang1zhi1li4* 'with all one's might'	0.975103162
神马都是浮云 *shen2ma3 dou4shi4 fu2yun2* 'all is vanity'	0.875783117
土豪 *tu3hao2* 'tuhao, rich man	0.929814503
非诚勿扰 *fei1cheng2 wu4rao3* 'Serious Suitors Only (the most-viewed dating show in China)'	0.952361166
奇葩 *qi2pa1* 'oddball'	0.970312506
表情帝 *biao3qing2di4* 'walking emoji'	0.990018764
倒逼 *dao4bi1* 'backfire'	0.967640631
二代 *er4dai4* 'scion'	0.884161842
断崖 *duan4ya2* 'sharp drop off'	0.863011143
Mean value	0.8975
